# *De Novo* characterization of the banana root transcriptome and analysis of gene expression under *Fusarium oxysporum* f. sp. *Cubense* tropical race 4 infection

**DOI:** 10.1186/1471-2164-13-650

**Published:** 2012-11-21

**Authors:** Zhuo Wang, JianBin Zhang, CaiHong Jia, JuHua Liu, YanQiang Li, XiaoMin Yin, BiYu Xu, ZhiQiang Jin

**Affiliations:** 1 Key Laboratory of Tropical Crop Biotechnology, Ministry of Agriculture, Institute of Tropical Bioscience and Biotechnology, Chinese Academy of Tropical Agricultural Sciences, Hainan, 571101, China; 2 Haikou Experimental Station, Chinese Academy of Tropical Agricultural Sciences, Hainan, 570101, China; 3 College of Agriculture, Hainan University, Hainan, 570228, China

## Abstract

**Background:**

Bananas and plantains (Musa spp.) are among the most important crops in the world due to their nutritional and export value. However, banana production has been devastated by fungal infestations caused by *Fusarium oxysporum* f. sp. *cubense* (Foc), which cannot be effectively prevented or controlled. Since there is very little known about the molecular mechanism of Foc infections; therefore, we aimed to investigate the transcriptional changes induced by Foc in banana roots.

**Results:**

We generated a cDNA library from total RNA isolated from banana roots infected with Foc Tropical Race 4 (Foc TR 4) at days 0, 2, 4, and 6. We generated over 26 million high-quality reads from the cDNA library using deep sequencing and assembled 25,158 distinct gene sequences by *de novo* assembly and gap-filling. The average distinct gene sequence length was 1,439 base pairs. A total of 21,622 (85.94%) unique sequences were annotated and 11,611 were assigned to specific metabolic pathways using the Kyoto Encyclopedia of Genes and Genomes database. We used digital gene expression (DGE) profiling to investigate the transcriptional changes in the banana root upon Foc TR4 infection. The expression of genes in the Phenylalanine metabolism, phenylpropanoid biosynthesis and alpha-linolenic acid metabolism pathways was affected by Foc TR4 infection.

**Conclusion:**

The combination of RNA-Seq and DGE analysis provides a powerful method for analyzing the banana root transcriptome and investigating the transcriptional changes during the response of banana genes to Foc TR4 infection. The assembled banana transcriptome provides an important resource for future investigations about the banana crop as well as the diseases that plague this valuable staple food.

## Background

Bananas and plantains (*Musa* spp.), which are staple foods due to their high protein content and nutrition value as well as the main income source in many developing countries, are among the most important crops in the world. In fact, banana ranks as the fifth most important agricultural crop in world trade, making it the world’s leading fruit crop and a significant economic backbone to the export industry of many agriculture-based countries in Asia, Africa, and Latin America [1]. Therefore, the global and local health of banana crops is of utmost importance to the world economy.

There are several devastating diseases that target the *Musa* crop [2]. One such disease, Panama disease or Fusarium wilt, is caused by the fungus *Fusarium oxysporum* f. sp*. cubense* (Foc) [3] and is widely regarded as one of the most destructive plant diseases in the world. To date, Foc has devastated banana production and continues to threaten crops [4]. The disease was first reported in 1874 in Australia and later destroyed the export trade based on the variety ‘Gros Michel’ by the 1950s. Since the 1960s, the resistant ‘Cavendish’ (AAA) subgroup of cultivars has dominated banana exports, becoming the major commercial variety in the world. However, an extremely virulent form of Foc, called ‘Tropical Race 4’ (Foc TR4), is capable of attacking the susceptible Cavendish variety, causing large losses in banana production in recent years [5].

Foc infects the lateral or feeder roots of banana plants upon contact [6]. Foc infection causes wilt syndrome with the typical symptoms of necrosis and rotting of roots, rhizomes, and pseudostem vessels, which turn a reddish-brown/maroon color as the fungus grows through the tissues. After the decay of infected plants, the pathogen can survive in soil in chlamydospore form over a long period of time to infect other plants. Foc spores can spread through water or soil, and by adhering to vehicles and footwear. In the soil, Fusarium is difficult to control by general chemical measures, such as fungicides or soil fumigants [7]. Therefore, resistance breeding is the preferred method of overcoming the Fusarium wilt of banana plants. However, because Cavendish bananas have a triploid (AAA) genome, they do not produce seeds, which hinders conventional breeding strategies [2].

Genetic engineering methods can improve the disease resistance of banana plants to Fusarium wilt [8]; however, little is known about the actual transcriptional changes and their regulation during the pathogen-plant interaction. Understanding the underlying changes during this interaction would allow for the identification of signal transduction pathways affected by infection and the interaction mechanisms during infection, which can lead to improvement of disease resistance of the banana plants. Traditional genome-wide analysis of gene expression of organisms under different conditions or, in the case of pathogens, at different life cycle stages, has mainly been carried out by microarrays, suppression subtractive hybridization (SSH), and cDNA-AFLP methods [9-12]. Van den Berg *et al*. (2007) used SSH and microarrays to show that cell wall-strengthening genes may be important for banana resistance to Fusarium wilt. However, the approach that was used suffers a number of drawbacks, including the fact that the genes are far from complete with only 79 clones [10]. Recently, with the completion of banana genome sequence, a doubled haploid *M. acuminata* genotype (AA) has been shown to be highly resistant to Foc TR4 by phenotyping assays [13], but further research on its mechanism has not been performed, especially with relation to transcription. A resistant variety of the Cavendish banana (AAA) was acquired by somaclonal variation, using the RNA-seq and DGE methods, and it was discovered that recognition of PAMPs (pathogen-associated molecular pattern) and defense-related transcripts are involved in banana resistance to Foc TR4 infection [14]. Therefore, elucidation of the mechanism by which Cavendish bananas respond to Foc TR4 infection is imperative.

One such promising method developed in recent years is next-generation sequencing, by which an enormous amount of sequence data can be rapidly obtained within a short period of time due to its high-throughput and high-coverage nature [15,16]. RNA-Seq technology, which is based on deep-sequencing, enables more precise quantification of genome-wide transcript levels than previous, microarray-based methods [17]. In this technology, whole mRNA or cDNA is mechanically fragmented for deep-sequencing, the results of which can be then mapped on a reference genome or used in *de novo* assembly to obtain a genome-wide transcriptome. Another method of great value for expression analysis is digital gene-expression (DGE) [18]. DGE uses 17–21 base pair (bp) short fragments from the whole transcriptome as gene-specific tags and calculates the expression level of a gene from the frequency of its tag.

We previously used a green fluorescent protein (GFP)-tagged strain of Foc TR4 and characterized early events in infection and disease development of Cavendish plantlets [19]. The combination of DGE and RNA-Seq allows us to easily perform transcriptome analysis without the need for an already-assembled reference genome. Despite the importance of the Foc pathogen for global banana production, RNA-Seq and DGE have not been used to investigate the main questions underlying the pathogen-banana interaction. Therefore, we aimed to investigate the changes in gene expression during Foc TR4 infection of banana roots using RNA-Seq and DGE analysis. For this purpose, we generated over 2.39 billion bases of high-quality DNA sequence and demonstrated the suitability of short-read sequencing for assembly and annotation of genes expressed in a triploid-genome plant without previous whole-genome information. We then identified 25,158 distinct sequences. Furthermore, we compared the gene expression profiles during an infection time course using DGE analysis. The assembled and annotated gene expression profiles provide an invaluable resource for the identification of differentially expressed genes during Foc TR4 infection of banana, which will enable us to screen for host susceptibility factors and to monitor shifts in Foc TR4 virulence.

## Results and discussion

### Assembly of a high-quality banana root transcriptome

In the absence of a sequenced genome, *de novo* assembly of RNA-Seq data was the only viable option to study the banana transcriptome. To obtain an overview of the expression profile of banana roots under Foc TR4 stress, a cDNA sample was prepared from the total RNA of an equal mixture of roots not infected and infected with Foc TR4 for 2, 4, and 6 days to acquire the genes whose expression is specifically altered when the plant is infected by Foc TR4.

Deep-sequencing of this cDNA sample produced 26,662,006 sequence reads with a length of 90 bp each (including single-end reads and paired-end reads), which corresponded to approximately 2.39 gigabase pairs (Gbp) of raw data. An overview of the sequencing and assembly is outlined in Table 1.

**Table 1:** Summary for the banana root transcriptome

**Total number of reads**	
Total base pairs (bp)	2,399,580,540 bp
Average read length	90 bp
Total size of scaffolds	28,778,591 bp
Total number of scaffolds > 100 bp	25,158
Total number of scaffolds > 2 kb	5,166
Mean length of scaffolds	1439 bp
Longest scaffold length	12,963 bp

The raw reads were first assembled into a draft using SOAP *de novo*-Oases software [20], and further assembly was achieved using CAP3 Sequence Assembly Software. After assembling, reads were also mapped back to the assembled transcripts with a length ≥ 100 bp. If the coverage of two assembled reads was more than 80%, then the shorter one was eliminated. The remaining sequences were then assembled into 111,825 contigs (Table 1). The mean contig size was 259 bp with lengths ranging from 100 to 9,135 bp, including 697 contigs larger than 2,000 bp. A total of 102,439 contigs were confirmed using the banana EST library (http://esttik.cirad.fr/cgi-bin/public_download.cgi). The mean contig size in the final library was 281 bp with lengths ranging from 100 to 9,135 bp, including 728 contigs larger than 2,000 bp. Using paired-end joining and gap-filling, the contigs were further assembled into 25,158 scaffolds with a mean size of 1,439 bp, including 5,166 scaffolds larger than 2,000 bp. The longest scaffold was 12,963 bp (Table 1). To evaluate the quality of the dataset, we analyzed the gap-filling to assembled contigs length. The total size of all contigs was 28,778,591 bp with a total 7,327,512 bp gap size. The total size of the scaffolds was 36,106,103 bp. In order to evaluate our data, the assembled banana transcriptome was searched using BLASTn against plant cDNAs (*Arabidopsis* and Rice) using a cut-off E-value of 10^-10^. Using this approach, approximately 99.5% contigs (28,644,628 bp) aligned successfully to plant cDNA with a total gap size of 7,293,951 bp, which was 33,561 bp shorter than the banana gap size. In addition, 80.27% of the reads and 74.84% of the paired-end joined sequences could be mapped onto the banana transcriptome. Importantly, 90% of the distinct gene sequences were unique. Our results indicated that the banana root transcriptome was of high quality. Transcripts with lengths ≥ 100 bp were subsequently used for analysis.

### Functional annotation of the banana root transcriptome

**Figure 1:**
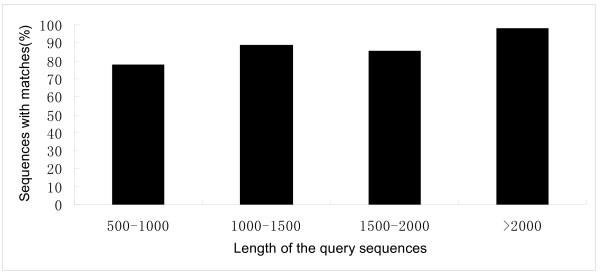
**Effect of query sequence length on the percentage of matching sequences.** The proportion of sequences with matches with a cut-off E-value of 10^-5^ in the NCBI NR database is greater for the longer assembled sequences.

We acquired 25,158 distinct gene sequences, 5,166 of which were longer than 2,000 bp. Compared with the 15,464 EST and 2,937 nucleotide sequences in NCBI database of banana, our data enriched the gene resources for banana. To annotate, classify, and functionally map the 25,158 distinct gene sequences, we used BLASTx to match the distinct gene sequences using a cut-off E-value of 10^-5^, including the non-redundant protein database (NR, NCBI), Gene Ontology (GO), and the Kyoto Encyclopedia of Genes and Genomes (KEGG) database with a cut-off E-value of 10^-5^. Using this approach, 21,622 distinct gene sequences (85.94% of all distinct gene sequences) returned a valid BLAST result (Additional file 1), confirming the high quality of our transcriptome assembly. Fourteen percent (3,536) of the distinct gene sequences could not be matched to known genes. To annotate the distinct gene sequences that we identified in our transcriptome assembly, we initially searched against the plant proteins in the NR database. As a result, we obtained 21,475 significant BLAST hits (85.36% of all distinct gene sequences, Table 2 and Additional file 1), which confirmed that most genes could be annotated after assembly. Interestingly, 77.4% of the 500 – 1,000 bp query sequences, 88.5% of the 1,000 – 1,500 bp, 85% of the 1,500 – 2,000 bp, and 98.8% of the query sequences longer than 2,000 bp were annotated successfully (Figure 1). This result indicated that the longer sequences provided more accurate matches with the NR database, while the shorter sequences lacked sufficient gene information to match, despite being easier to annotate.

**Table 2:** Function annotation of the banana roots transcriptome

		**Number**	**Percent (%)**
Total		21,622	85.94
Annotated	Nr	21,475	85.36
	GO	17,540	69.72
	Kegg	11,611	46.15

### GO classification

**Figure 2:**
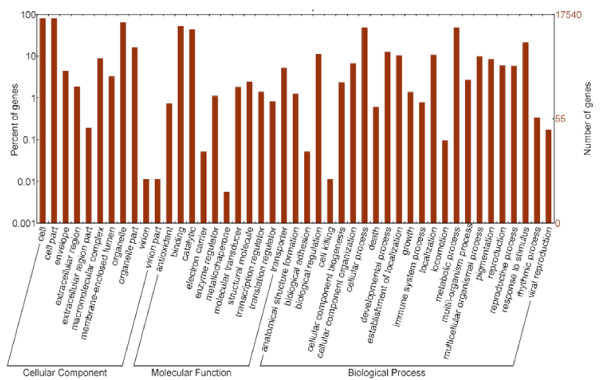
**Histogram of GO classifications.** The results are summarized in three main GO categories: biological process, cellular component, and molecular function. The left y-axis indicates the percentage of a specific sub-category of genes in that main category, the right y-axis indicates the number of genes in a sub-category.

Out of the 21,475 annotated distinct gene sequences, 17,540 (69.72%) were assigned 10,428 GO terms using BLAST2GO. Forty-five GO sub-categories were represented under three major categories (Figure 2).

The majority of the GO annotations were in the cellular component category, assigned to 4,157 (23.7%) distinct gene sequences, followed by the biological process category for 3,701 (21.1%) distinct gene sequences, and the molecular function category for 9,682 (55.2%) distinct gene sequences. The major sub-categories are shown in Figure 2: four major cellular component sub-categories were “cell” (GO: 0005623), “cell part” (GO: 0044464), “organelle” (GO: 0043226), and “organelle part” (GO:0044422); two major molecular functions sub-categories were “binding” (GO: 0005488) and “catalytic” (GO: 0003824); and four major biological process sub-categories were “metabolic process” (GO: 0008152), “cellular process” (GO: 0009987), “metabolic process” (GO: 0008152), and “response stimulus” (GO:0050896). However, only 69.72% of banana root distinct gene sequences were assigned with these GO terms, possibly because the large number of uninformative gene descriptions of these plant protein hits.

### Kyoto encyclopedia of genes and genomes (KEGG) pathway mapping

By mapping EC numbers to the reference canonical pathways, 11,611 (46.15%) distinct gene sequences were assigned to 192 KEGG pathways. The pathways most represented by unique sequences were carbohydrate metabolism (1,448 members), amino acid metabolism (978), signal transduction (921), and cell growth and death (787). Taken together, these annotations provide a valuable resource for investigating the specific processes, structures, functions, and pathways involved in the response to the infection of Foc TR4 in banana roots.

### Statistics of DGE tags

Using the DGE method, which generates absolute rather than relative gene expression measurements and avoids many of the inherent limitations of microarray them with analysis, we analyzed the gene expression profile of banana roots after inoculating Foc TR4. Total RNA isolated from banana roots at 0, 2, 4, and 6 days post-inoculation (DPI) were analyzed by Illumina DGE tag profiling to create transcriptome profiles of the four groups. DGE tags were derived from the 3’UTR of transcripts and were 21-nucleotides long. DGE data provided a quantitative measure of transcript abundance in the RNA population. DGE analysis also allowed for the identification of previously unannotated genes. The majority of DGE tags were expected to match only one location in the genome, with the remaining tags matching duplicate genes, alternate transcripts, antisense strands, or repeated sequences [21].

**Figure 3:**
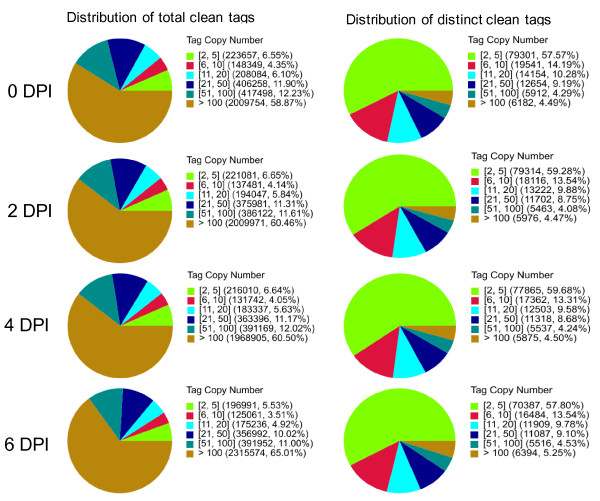
**Distribution of total tags and distinct tags over different tag abundance categories.** (A) Distribution of total tags. Numbers in the square brackets indicate the range of copy numbers for a specific category of tags. For example, [2,5] means all the tags in this category had 2 – 5 copies. Numbers in the parentheses show the total tag copy number for all the tags in that category. (B) Distribution of distinct tags. Numbers in the square brackets indicate the range of copy numbers for a specific category of tags. Numbers in the parentheses show the total types of tags in that category.

We obtained a total of 3,570,000, 3,521,001, 3,790,500, and 3,500,000 total tags and 366,382, 384,048, 335,285, and 297,960 distinct tags from the roots of the 0, 2, 4, and 6 DPI time points, respectively. Heterogeneity and redundancy are two significant characteristics of mRNA expression, and while the majority of mRNAs are expressed at low levels, a small proportion is highly expressed. Therefore, the distribution of tag expression was used to evaluate the normality of the DGE data. As shown in Figure 3, the distribution of total tags and distinct tags over different tag abundance categories showed similar patterns for all four DGE libraries.

### Mapping sequences to the reference transcriptome database

To identify the molecular events behind Foc TR4 infection of banana roots, we mapped the tag sequences of the four DGE libraries to our transcriptome reference database. Among the 121,777 – 137,744 distinct tags generated from the Illumina sequencing of the four libraries, 18161 – 21,661 distinct tags were mapped to a gene in the reference database (Table 3). Tags mapped to a unique sequence are the most critical subset in DGE libraries, as they can explicitly identify a transcript. Up to 14.44% (18,161) of the sequences in our transcriptome reference tag database could be unequivocally identified by a unique tag (Table 3).

**Table 3:** Summary of DGE sequencing results

**Summary**		**0 DPI**	**2 DPI**	**4 DPI**	**6 DPI**
Raw Data	Total	3,570,000	3,521,001	3,790,500	3,500,000
Raw Data	Distinct Tag	366,382	384,048	335,285	297,960
All Tag Mapping to Gene	Total number	1,026,297	1,018,511	1,044,196	946,905
All Tag Mapping to Gene	Total % of clean tag	30.87%	31.29%	29.32%	28.56%

To determine whether our DGE tags reached saturation, we compared the increase in the distinct tag number to the increase in total tag number. When sequencing depths reached 2 million or more base pairs, the number of distinct tags discovered almost ceased to increase in all four libraries, which indicated that the sequencing was saturated (Additional file 2).

The level of gene expression was then determined by calculating the number of unambiguous tags for each distinct gene sequence and then normalizing this to the number of transcripts per million tags (TPM). Additional file 3 provides a list of the top 20 most abundantly expressed genes in the 2 DPI library as an example. Comparing our results with those of Van Den Berg (2007), the expressions of two catalases, two pectin acetyl esterases and three pathogenesis-related proteins in our result in the 2 DPI library were consistent. The result indicated that those genes responded to Foc TR4 infection [10].

### Gene expression profile changes in banana roots infected with Foc TR4

**Figure 4:**
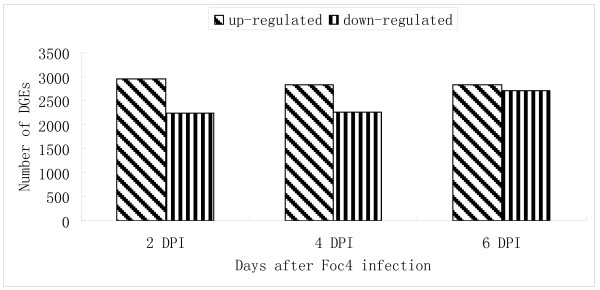
**Changes in gene expression profile of banana roots with the progression of the Foc TR4 infection.** The numbers of up- and down-regulated genes in 2, 4, and 6 DPI compared to 0 DPI are summarized.

To identify the signaling pathways involved in the banana response to Foc TR4 infection, we identified tags that were differentially expressed between the 0 DPI and the later infection time points using an algorithm developed by Audic *et al*. [22]. A total of 4,729 distinct gene sequences significantly changed between the 0 and 2 DPI libraries, where 2,496 distinct gene sequences were upregulated and 2,233 distinct gene sequences were downregulated after 2 days of Foc TR4 infection. Between the 0 and 4 DPI libraries, a total of 5,078 distinct gene sequences were detected with 2,825 upregulated distinct gene sequences and 2,253 downregulated gene sequences. There were 5,531 distinct gene sequences that were expressed at a different level in the 0 and 6 DPI libraries, with 2,821 upregulated distinct gene sequences and 2,710 downregulated distinct gene sequences after 6 days of infection (Figure 4).

Gene ontology analysis was used for the above differential expression distinct gene sequences, and enrichment analysis was performed using a false discovery rate (FDR) adjusted p-value of ≤0.05 as the cutoff. The downregulated distinct gene sequences did not enrich any GO term, while the upregulated distinct gene sequences enriched 8, 22, and 11 featured GO terms at 2 DPI, 4 DPI and 6 DPI respectively (Additional file 4). In particular, response to stress (GO:0006950) was enriched at 4 DPI and response to chemical stimulus (GO:0042221) was enriched at 6 DPI, which suggested that banana root was subjected to stress and chemical stimulation because of Foc TR4 infection at these two time points.

Although down-regulated expression of distinct gene sequences did not enrich the GO term, we did find that some distinct gene sequences had down-regulated expression, such as nsp-interacting kinase [23] and sumo E3 protein ligase [24]. These genes were included in the immune-related GO term, which indicated that those distinct gene sequences did not respond to Foc TR4 infection.

### KEGG pathway analysis of differentially expressed banana roots genes in response to infection by Foc TR4

To understand the functions of differentially expressed distinct gene sequences, we mapped them to KEGG terms to discover those genes involved in metabolic or signal transduction pathways that were significantly enriched. Additional file 5 shows enriched pathways at 2 DPI, 4 DPI and 6 DPI. Phenylalanine metabolism was enriched at 2 DPI, 4 DPI, and 6 DPI (Additional file 5). There are twenty-three peroxidases, twelve bacterial-induced peroxidase precursors, five 4-coumarate: coenzyme a ligases, three cinnamate 4-hydroxylases and three phenylalanine ammonia lyases enriched in this pathway. Meanwhile, 17 peroxidases, four 4-coumarate:coenzyme a ligases, two cinnamate 4-hydroxylases and one phenylalanine ammonia lyase were enriched in Phenylpropanoid biosynthesis at 2 DPI. It should be emphasized that peroxidases were enriched in both pathways. The peroxidases enriched in those pathways may be involved in increased lignin biosynthesis [25], and may acting as basal defense components: peroxidase is one source for the production of ROS [26]. That the peroxidases were upregulated suggests that banana roots responded to infection by Foc TR4 by ROS production. Similarly, enrichment of drug metabolism-cytochrome P450 was found at 2DPI. There are 10 distinct gene sequences of Glutathione S-transferases (GSTs, E.C.2.5.1.18) or glutathione transferases in this pathway (Additional file 6). GSTs, as a heterogeneous group of cell detoxifying enzymes, catalyse the conjugation of tripeptide glutathione (GSH) to electrophilic sites on a wide range of phytotoxic substrates [27,28]. It is likely that even the susceptible cultivar activates some early mechanisms of defense against Foc TR4; however, these are not sufficient to provide resistance against the pathogen.

At 6 DPI, alpha-linolenic acid metabolism was enriched, leading to jasmonic acid biosynthesis, which is one of the pathways associated with pathogen resistance and the genes in this pathway were significantly affected by Foc TR4 infestation at all time points (Additional file 7). This is consistent with previous reports that biotic and abiotic stresses, such as pathogen infection, wounding and insect feeding, can trigger JA biosynthesis through direct activation of genes encoding the relevant biosynthetic enzymes [29]. Ethylene and SA biosynthetic and signaling related genes showed no significant differences between non-inoculation and inoculation in our results. These results indicate that JA biological synthesis may be regulated by Foc TR4 infection. Similarly, the expressions of JA biosynthetic and signaling related genes in a resistant variety were higher than in a susceptible variety [14]. Further study of these genes in this pathway could identify them as targets for testing whether a variety is resistant to Foc TR4 infestation.

## Conclusions

Here, we present a rapid and low-cost method for triploid plant transcriptome assembly and DGE analysis using Illumina sequencing technology. Our findings provide a substantial contribution to the existing sequence resources for the banana and will certainly accelerate research regarding the devastating Foc TR4 pathogen of this valuable staple food. Our expression analysis results provide promising leads for future functional studies for understanding how the Foc TR4 pathogen infects and kills banana plants.

## Methods

### Plant materials and treatments

Banana plantlets (*Musa acuminata* L. AAA group*,* ‘Brazilian’) were obtained from the Tissue Culture Center of Chinese Academy of Tropical Agricultural Sciences. The plants (1 plant/pot) were distributed at random in a glass greenhouse. The maximum and minimum temperatures in the greenhouse during the experiment were 30°C and 20°C, respectively, while relative humidity oscillated between 55% and 80%. Our previous study confirmed that the growth characteristics and virulence of GFP-tagged Foc TR4 did not change and that it could efficiently infect banana plants thereby inducing disease symptoms [19]. Once the plants had reached the five-leaf stage and developed a healthy root system (approximately 60 days), their roots were dipped in a Foc TR4 spore suspension of 1.5 × 10^6^ condia/mL. The entire root system was harvested at 0, 2, 4 and 6 days post-infection (DPI), flash-frozen in liquid nitrogen, and stored at −70°C. Ten plants were used for each time point. The roots of the uninfected banana plants were harvested at 0 day as described above.

### RNA extraction

Total RNA was extracted from the 0, 2, 4, and 6 DPI roots at the same time as described by Wan [30]. RNA integrity was confirmed using the 2100 Bioanalyzer (Agilent Technologies). All samples had a minimum RNA integrity (RIN) value of 8.20 μg of total RNA (a mixture of RNA from roots not infected and that infected with Foc TR4 for 2, 4, and 6 days at an equal ratio) was prepared for Solexa sequencing. Magnetic beads with polyT oligos attached were used for purifying the mRNA from the total RNA. The mRNA was then cleaved into small fragments with divalent cations at elevated temperature. The fragments were used to synthesize first-strand cDNA using random hexamer adapters and reverse transcriptase (Invitrogen, USA). This was followed by second-strand cDNA synthesis using DNA polymerase I (NEB, USA) and RNaseH (Invitrogen, USA). These cDNA fragments then went through an end repair process and were ligated to adapters. The final products were purified and enriched by PCR to create the final cDNA library.

### Analysis of illumina sequencing results

The cDNA library was sequenced on the Illumina GAII sequencing platform. The average read size of the library was approximately 200 bp and both ends of the cDNAs were sequenced. Image deconvolution and quality value calculations were performed using the Illumina GA pipeline 1.3. Sequences from the Illumia sequencing were deposited in the GenBank Short Read Archive (Accession number: SRA055079).The raw reads were cleaned by removing adapter sequences, empty reads, and low quality sequences (reads with unknown base pairs ‘N’). The reads obtained were randomly clipped into 21 bp K-mers for assembly using de Bruijn graph and SOAPdenovo software [20]. After assessing different K-mer sizes, we found that the 21-mer provided the best result for transcriptome assembly. Small K-mers resulted in graphic outputs that were too complex to be meaningful, while large K-mers resulted in poor overlap in regions with low sequencing depth. After sequence assembly, the resulting contigs were joined into scaffolds using the read-mate pairs. To obtain distinct gene sequences, the scaffolds were clustered using TGI Clustering tools [31]. Distinct sequences were used for BLAST search and annotation against the NCBI NR database using an E-value cut-off of 10^-5^. Functional annotation by gene ontology (GO, http://www.geneontology.org) terms was analyzed by BLAST2GO software (NCBI) [32]. The KEGG pathway annotation was performed using BLASTALL software (NCBI) [33]. The GeneID of the assembled sequences are provided in Additional file 1.

### DGE library preparation and sequencing

Tag library preparation for the different time points after Foc TR4 infection (0, 2, 4, and 6 DPI) was performed in parallel. Briefly, mRNA was captured with magnetic oligo (dT) beads from total RNA of banana roots infected with Foc TR4 for 0, 2, 4, or 6 days. First- and second-strand cDNAs were synthesized, and bead-bound cDNAs were subsequently digested with *Nla*III. The cDNA fragments with 3’ ends were then purified with magnetic beads and the Illumina adapter 1 was added to their 5’ ends. The junction of the Illumina adapter 1 and CATG site is the recognition site of *Mme*I, which cuts 17 bp downstream of the CATG site, producing tags with adapter 1. After removing the 3’ fragments with magnetic beads, Illumina adapter 2 was introduced at the 3’ end of tags, producing tags with different adapters at each end in the resulting tag library. After 15 cycles of linear PCR amplification, 85-base strips were purified by polyacrylamide gel electrophoresis. These strips were then digested, and the resulting single-chain molecules were fixed onto the Illumina sequencing chip for sequencing. The reproducibility of DGE was > 0.99 [34]. The data sets are available at the NCBI Short Read Archive with the accession number: SRX156204, SRX156205, SRX156206 and SRX156207.

### Analysis and mapping of DGE tags

The raw image data obtained from sequencing was transformed by base calling into sequence data. Before mapping the reads to the reference database, we filtered all sequences to remove adaptor sequences, low quality sequences, empty tags (sequences with only adaptor sequences), and tags with a copy number of 1 (probably resulting from sequencing errors). A preprocessed database of all possible CATG+17-nucleotide tag sequences was created using our transcriptome reference database. For annotation, all tags were mapped to the reference sequences and only 1 nucleotide mismatches was allowed. All the tags mapped to reference sequences from multiple genes were filtered and the remaining tags were designated as unambiguous tags. For gene expression analysis, the number of expressed tags was calculated and normalized to the number of transcripts per million (TPM) tags. The differentially expressed tags were used for further mapping and annotation.

### Evaluation of DGE libraries

A statistical analysis of the frequency of each tag in the cDNA libraries from the 0, 2, 4, and 6 DPI samples was performed to compare gene expression during the infection time course using the method described by Audic *et al*. [23]. FDR was used to determine the threshold of the p-value in multiple tests and analyses. We used an FDR < 0.001 as the threshold to judge the significance of gene expression differences. For pathway enrichment analysis, we mapped all differentially expressed genes to KEGG pathway terms and identified significantly enriched KEGG terms compared with the assembled transcriptome background.

## Competing interests

The authors declare that they have no competing interests.

## Authors’ contributions

The study was conceived by WZ, ZJB, XBY and JZQ. The plant material preparation and Foc TR4 management were carried out by WZ, ZJB, LJH, JC and YXM. LYQ contributed to the data analysis, bioinformatics analysis. WZ, XBY and JZQ contributed to manuscript preparation. All authors have read and approved the final manuscript.

## Supplementary Material

Additional file 1 Gene ID and expression of the assembled sequences. Click here for file

Additional file 2 **Relationship between the number of detected genes and sequencing amount (total tag number).** All figures show a trend of saturation. When the sequencing amount reaches 2 millions, the number of detected genes almost ceases to increase. Click here for file

Additional file 3 **Summary of the most abundant genes expressed in 2 DPI with annotation.** TPM: number of transcripts per million tags. Click here for file

Additional file 4 GO term are enriched in 2 DPI, 4 DPI and 6 DPI. Click here for file

Additional file 5 Pathways are enriched in 2 DPI, 4 DPI and 6 DPI. Click here for file

Additional file 6 Genes are enriched in Drug metabolism - cytochrome P450. Click here for file

Additional file 7 Gene expression of alpha-linolenic acid metabolism pathways in 2 DPI, 4 DPI and 6 DPI. Click here for file
