# Supplementary material for: De Novo characterization of the banana root transcriptome and analysis of gene expression under Fusarium oxysporum f. sp. Cubense tropical race 4 infection
Source: BMC Genomics. 2012 Nov 21;13:650. doi: 10.1186/1471-2164-13-650 (PMC3534498; doi:10.1186/1471-2164-13-650)

Additional file 2 - Distribution of total tags and distinct tags over different tag abundance categories. All figures show a trend of saturation. When the sequencing amount reaches 2 millions, the number of detected genes almost ceases to increase.

0 DPI


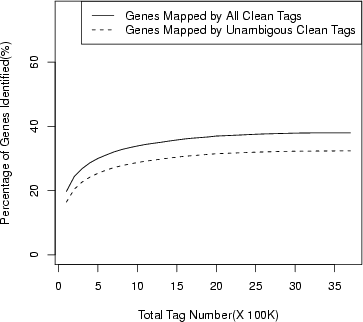


2 DPI


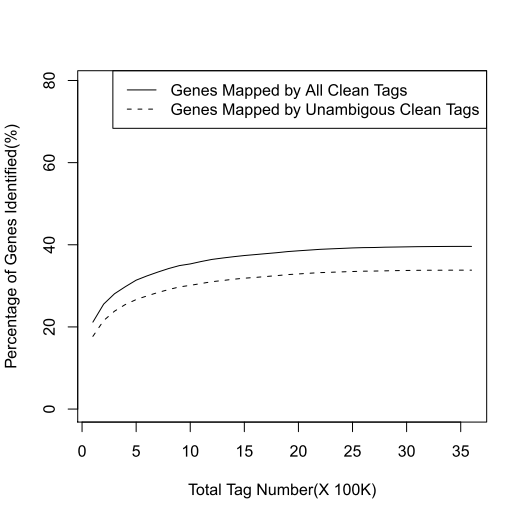


4 DPI


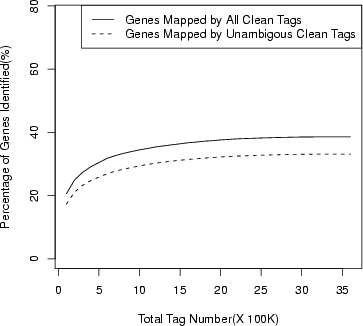


6 DPI


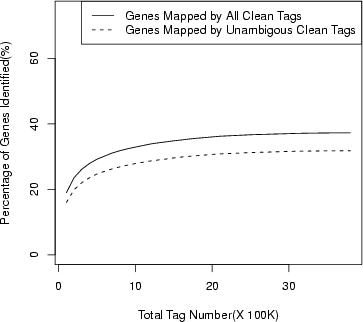

Supplement: Additional file 2 — Relationship between the number of detected genes and sequencing amount (total tag number). All figures show a trend of saturation. When the sequencing amount reaches 2 millions, the number of detected genes almost ceases to increase. [file 1471-2164-13-650-S2.doc]
